# PHOTH-graphene: a new 2D carbon allotrope with low barriers for Li-ion mobility

**DOI:** 10.1038/s41598-024-59858-y

**Published:** 2024-04-25

**Authors:** E. A. J. Santos, K. A. L. Lima, F. L. L. Mendonça, D. A. da Silva, W. F. Giozza, L. A. Ribeiro Junior

**Affiliations:** 1 Institute of Physics, University of Brasília, 70910-900 Brasília, Brazil; 2 Computational Materials Laboratory, LCCMat, Institute of Physics, University of Brasília, 70910-900 Brasília, Brazil; 3 Department of Electrical Engineering, Faculty of Technology, University of Brasília, Brasília, Brazil; 4 Professional Postgraduate Program in Electrical Engineering - PPEE, University of Brasília, Brasília, Brazil

**Keywords:** 2D Carbon allotrope, Graphynes, PHOTH-Graphene, Density functional theory, Condensed-matter physics, Nanoscale materials, Theory and computation, Graphene

## Abstract

The continued interest in 2D carbon allotropes stems from their unique structural and electronic characteristics, which are crucial for diverse applications. This work theoretically introduces PHOTH-Graphene (PHOTH-G), a novel 2D planar carbon allotrope formed by 4-5-6-7-8 carbon rings. PHOTH-G emerges as a narrow band gap semiconducting material with low formation energy, demonstrating good stability under thermal and mechanical conditions. This material has slight mechanical anisotropy with Young modulus and Poisson ratios varying between 7.08-167.8 GPa and 0.21-0.96. PHOTH-G presents optical activity restricted to the visible range. Li atoms adsorbed on its surface have a migration barrier averaging 0.38 eV.

## Introduction

Exploring new carbon-based materials^1,2^ has been a focal point of scientific research on nanomaterials, driven by the extraordinary properties of 2D carbon allotropes^3,4^, such as graphene^5^. This material comprises a single layer of carbon atoms arranged in a hexagonal lattice. It has emerged as a paradigm-changing material with exceptional electrical, thermal, and mechanical characteristics^6,7^. The unique properties of graphene have spurred various innovative applications, such as those related to energy conversion and storage^8^.

In the ever-evolving landscape of 2D carbon allotropes^9,10^, recent breakthroughs have expanded the repertoire beyond the renowned graphene. The synthesis of the biphenylene network^11^, gamma-graphyne^12^, monolayer amorphous carbon^13,14^, and monolayer fullerene network^15,16^ has broadened the class of reliable 2D carbon-based materials, pointing to new trends in flat electronics. In particular, the biphenylene network (BPN), characterized by interconnected rings of 4, 6, and 8 atoms, introduces a novel honeycomb-like structure that emerges as a variant of graphene and showcases promising electronic and mechanical properties^11^. Its regularly spaced pairs of biphenylene units contribute to its unique structural^17–19^ and optoelectronic^20,21^ properties, rendering it suitable for applications in lithium-ion batteries^22,23^. Importantly, its synthesis route opens channels for the fabrication and theoretical prediction of other allotropes that can combine these types of rings.

Incorporating porosity into 2D carbon-based nanomaterials introduces compelling features into their structures, rendering diverse applications^24,25^. Usually, materials endowed with rings of reasonable diameters provide high surface areas, making them candidates for gas adsorption, storage, separation, and catalysis^26,27^. In electrochemical energy storage, for example, the porous nature improves active sites, improving performance in batteries and supercapacitors^28^. In this way, the focus on seeking 2D carbon materials endowed with rings of specific diameters to facilitate permeability represents a strategic approach to material design. This study is situated within this context and aims to suggest a new 2D carbon allotrope where manipulated ring diameters are critical to unlocking enhanced ionic and small-molecule adsorptions.

**Figure 1:**
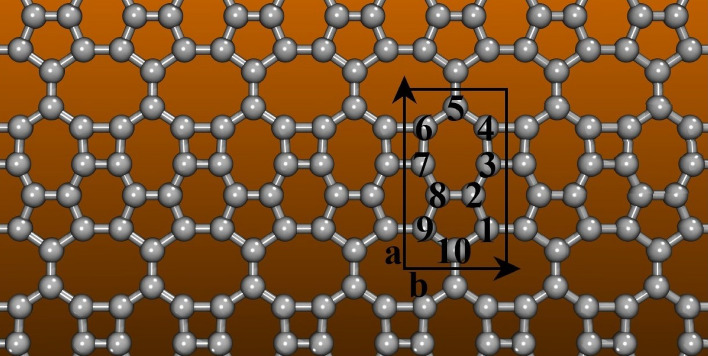
The lattice topology of PHOTH-G. The unit cell and related lattice vectors are highlighted in black. The grey spheres and sticks denote the carbon atoms and bonds between them. This figure was prepared using the Visual Molecular Dynamics software (VMD^30^, refer to https://www.ks.uiuc.edu/Research/vmd/).

Herein, we employed a numerical protocol to introduce a new 2D carbon allotrope denoted as PHOTH-Graphene (PHOTH-G, depicted in Fig. 1), obtained through a bottom-up approach. The combination of 4-5-6-7-8 membered rings of carbon atoms forms this allotrope. Its electronic, optical, and mechanical properties were investigated using density functional theory (DFT) and ab initio molecular dynamics (AIMD) simulations. PHOTH-G exhibits metallic behavior and structural stability, maintaining integrity even under the demanding conditions of 1000K AIMD simulations. This material displays optical activity across the visible spectrum. PHOTH-G showcases a low average diffusion barrier of approximately 0.38 eV for Li atoms.

## Results

We present the structural characteristics of PHOTH-G in Fig. 1. Consistent lattice parameters were achieved by optimizing the structure utilizing both DFT methods. The results of lattice optimization highlight the values explicitly obtained within the PBE scheme. The PHOTH-G lattice comprises 4, 5, 6, 7, and 8-membered rings, forming a planar structure without observed buckling. The unit cell with related lattice vectors ($a=3.89$ Å and $b=6.87$ Å), highlighted in black, consists of 10 atoms. Table 1 details the bond distances between these atoms. The bond lengths observed in PHOTH-G are similar to the ones identified in other flat carbon-based materials^29^, emphasizing the structural consistency of the studied material.

The PHOTH-G lattice shows an orthorhombic arrangement within the *P*1 space group, with bond distances falling within the 1.410–1.435 Å range. The formation energy of PHOTH-G is approximately −8.27 eV/atom, a value lower than other recently predicted 2D carbon allotropes^29^ and closely aligned with BPN (−7.4 eV/atom)^20,21,29^ and graphene (−8.8 eV/atom)^29,31^. PHOTH-G exhibits a planar density of 0.37 atom/Å^2^ (similar to BPN, about atom/Å^2^^21^), surpassing both graphene and graphdiyne (0.29 atom/Å^2^^32^ and 0.23 atom/Å^2^^33^, respectively). Moreover, it falls short of the density observed in DHQ-graphene and graphene (0.33 atom/Å^2^^34^ and 0.38 atom/Å^35^, respectively).

**Table 1:** PHOTH-G Bond lengths between the atoms labeled in Fig. 1.

**Bond type**	**Bond length (Å)**	**Bond type**	**Bond length (Å)**
C1–C2	1.418	C7–C8	1.358
C2–C3	1.346	C8–C9	1.407
C3–C4	1.420	C9–C10	1.457
C4–C5	1.407	C1–C10	1.442
C5–C6	1.386	C2–C8	1.360
C6–C7	1.418	–	–

**Figure 2:**
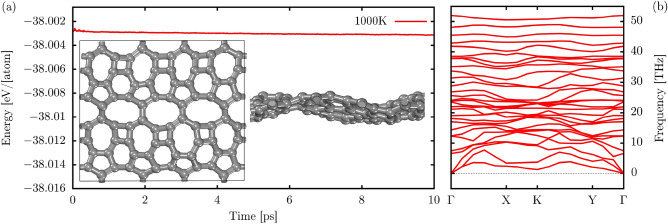
(**a**) Evolution profile of the total energy per atom lattice at 1000 K. The inset panels illustrate AIMD snapshots of PHOTH-G at 10 ps. (**b**) The phonon band structure of this material was computed using the PBE level.

AIMD simulation for the PHOTH-G lattice at 1000 K over 10 ps illustrated in Fig. 2a reveals the good thermal stability of PHOTH-G. A 3$\times$3$\times$1 supercell with 80 atoms was considered in these simulations. This set of parameters was also employed in other AIMD-based studies^36,37^. The total energy experiences minimal fluctuations during this timeframe, indicating the material’s robust behavior under reasonably high-temperature conditions. Detailed side and front views of the final AIMD snapshot (inset panels) depict an intact lattice structure with no observable bond breaking or reconstructions, underscoring the good thermal stability of PHOTH-G. At 1000 K, the final configuration aligns closely with the optimized structure, even with considerable in-plane and out-of-plane lattice distortions induced by thermal fluctuations.

The phonon dispersion curves of PHOTH-G are presented in Fig. 2b, which also provides information on its dynamical and thermal properties. The absence of phonon modes with imaginary frequencies suggests the inherent dynamic stability of PHOTH-G. The lack of a distinct band gap between acoustic and optical modes indicates a scattering rate and relatively shorter phonon lifetimes, contributing to the material’s moderate lattice thermal conductivity. Typically, stronger chemical bonds are associated with higher phonon frequencies. Notably, in the context of PHOTH-G, the highest frequency is approximately 52.0 THz, slightly surpassing that of graphene at 49.11 THz^38^, and aligning closely with the frequency reported for BPN^20^. This observation suggests the presence of stiff bonds within the fused 4-6-atom rings of PHOTH-G and BPN, constraining atomic oscillations compared to the more freely oscillating atoms in graphene.

**Figure 3:**
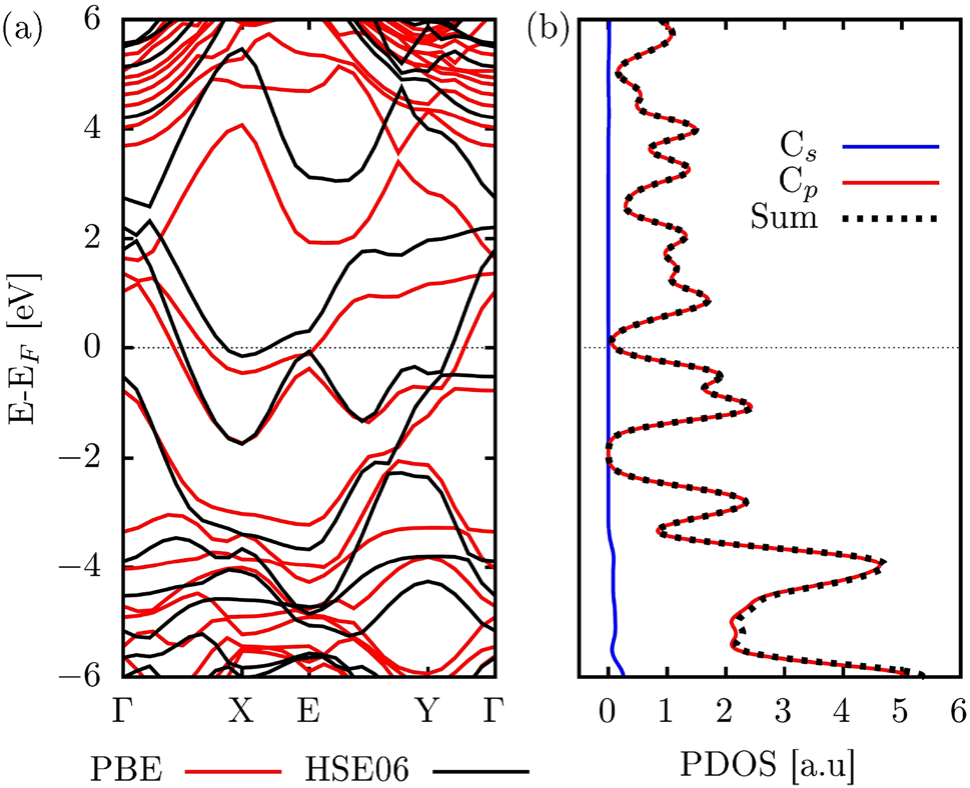
(**a**) The electronic band structure and (**b**) partial density of states (PDOS) for PHOTH-G are presented. The band structure was computed using both PBE (depicted in red) and HSE06 (depicted in black) methods, whereas PDOS calculations were specifically performed at the HSE06 level.

Next, we discuss the electronic properties of PHOTH-G. Fig. 3a presents the band structures of PHOTH-G computed at both the PBE and HSE06 levels. Both approaches consistently indicate a metallic signature for PHOTH-G, highlighting this material’s intrinsic free-like electronic conductivity. The slight discrepancy in the energy dispersion between PBE and HSE06 is characteristic of the tendency of PBE calculations to underestimate energy level values. At the same time, HSE06 typically provides more accurate descriptions of electronic and optical properties. Despite this, both methods closely align, reinforcing the inherent metallic trend observed in PHOTH-G.

In Fig. 3b, a comprehensive insight into the electronic structure of PHOTH-G at the HSE06 level is provided through the partial density of states (PDOS). The PDOS reveals the distinct involvement of various atomic orbitals in shaping the material’s electronic characteristics, with p-states playing a dominant role. These p-orbitals significantly influence electronic transitions and interactions within PHOTH-G, showcasing directional bonding phenomena. In contrast, s-states make a minor contribution to the valence levels, underscoring the relevance of p-orbitals in determining the electronic behavior of the material. The evident disparity in PDOS highlights PHOTH-G as a metallic substance.

**Figure 4:**
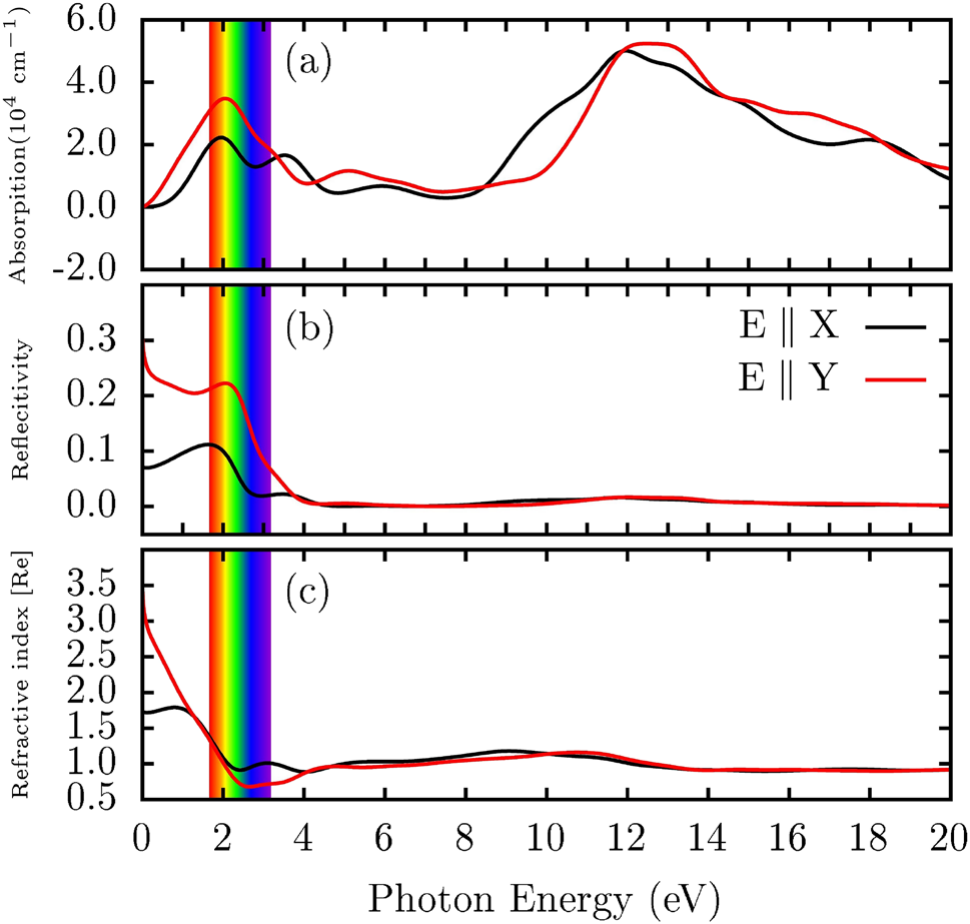
The optical characteristics of PHOTH-G are presented as a function of photon energy, encompassing (**a**) absorption coefficient, (**b**) reflectivity, and (**c**) refractive index.

We now delve into the optical properties of PHOTH-G. In Fig. 4a, the light absorption characteristics of PHOTH-G are depicted, unveiling fundamental optical attributes influenced by its lattice topology. The material demonstrates a reasonable absorption coefficient (10^4^ cm^-1^), indicative of its metallic nature. The initial absorption peaks for light polarized along the x (E//X) and y (E//Y) directions fall within the visible spectrum, in contrast to observations in graphene^38^. Specifically, the peak around 2.0 eV (in the red region of the visible spectrum) exhibits a red-shift of approximately 2.0 eV and 1.0 eV when compared to graphene^38^ and biphenylene^20,39^, respectively. Both graphene and biphenylene have initial peaks in the UV region, at around 4.0 eV^38^ and 4.5 eV^20,39^, respectively.

In Fig. 4b, PHOTH-G consistently maintains reflectivity coefficients below 0.3, indicative of effective transmission of incident light with minimal disruption. Notably, the highest reflectivity is observed within the visible spectrum, with peaks occurring around photon energies of 2.0 eV. This observation highlights the material’s ability to reflect incident light in the visible spectrum, aligning with similar behaviors observed in graphene^38^ and biphenylene^20,39^. The decreasing reflectivity peaks as energy increases may imply the presence of electronic resonances or specific vibrations in the material corresponding to these energy levels.

Figure 4c explores the refractive index of PHOTH-G, unveiling anisotropic characteristics influenced by its lattice structure. The material exhibits birefringence, where the speed of light varies in different polarization directions. Anisotropy in refractive indices is observed along polarization directions parallel to its basal plane, with the most notable refraction occurring at the infrared limit. This trend highlights the birefringent nature of PHOTH-G, distinguishing it from graphene. Moreover, there is a decline in the refractive index value, converging to 1.0 for photon energy values exceeding 4 eV. This convergence suggests uniform refraction of incident UV light in all directions.

The elastic properties of PHOTH-G, examining Poisson’s ratio ($\nu (\theta )$) and Young’s modulus ($Y(\theta )$) under pressure within the xy plane^40,41^ are

1 $$\begin{aligned} \displaystyle Y(\theta ) = \frac{{C_{11}C_{22} - C_{12}^2}}{{C_{11}\alpha ^4 + C_{22}\beta ^4 + \left( \frac{{C_{11}C_{22} - C_{12}^2}}{{C_{44}}} - 2C_{12}\right) \alpha ^2\beta ^2}} \end{aligned}$$

and

2 $$\begin{aligned} \displaystyle \nu (\theta )= \frac{{(C_{11} + C_{22} - \frac{{C_{11}C_{22} - C_{12}^2}}{{C_{44}}})\alpha ^2\beta ^2 - C_{12}(\alpha ^4 + \beta ^4)}}{{C_{11}\alpha ^4 + C_{22}\beta ^4 + \left( \frac{{C_{11}C_{22} - C_{12}^2}}{{C_{44}}} - 2C_{12}\right) \alpha ^2\beta ^2}}, \end{aligned}$$

**Figure 5:**
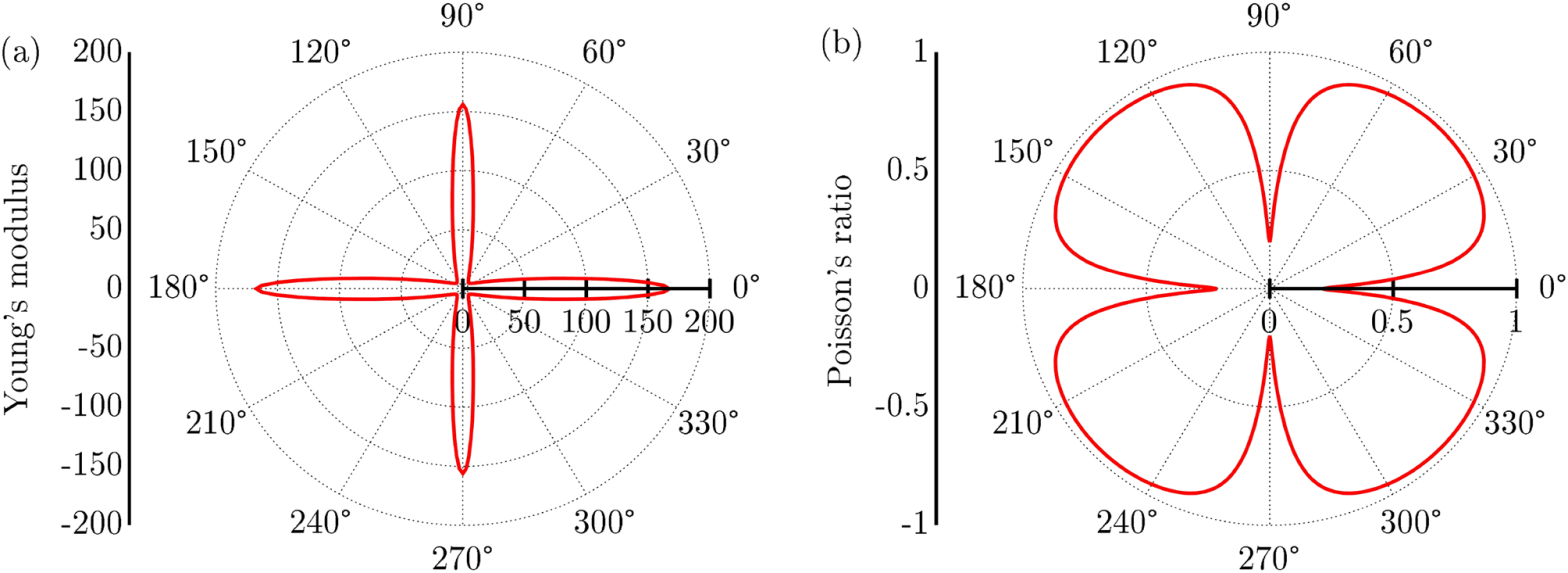
(**a**) Young’s modulus and (**b**) Poisson’s ratio regarding the basal plane of PHOTH-G.

Here, $\alpha =\cos (\theta )$ and $\beta =\sin (\theta )$. The elastic constants of PHOTH-G are meticulously presented in Table 2. Additionally, Figures 5a and b offer a 2D representation of Young’s modulus and Poisson’s ratio in the xy plane for this material.

**Table 2:** Elastic constants C_ij_ (GPa) and maximum values for Young’s modulus (GPa) ($Y_{MAX}$) and maximum ($\nu _{MAX}$) and ($\nu _{MIN}$) Poisson’s ratios.

Structure	C_11_	C_12_	C_22_	C_44_	$Y_{MAX}$	$\nu _{MAX}$	$\nu _{MIN}$
PHOTH-G	162.78	34.58	174.43	1.80	167.08	0.96	0.21

The elastic constants C11, C22, C12, and C44, detailed in Table 2, satisfy the Born-Huang criteria for an orthorhombic crystal ($C_{11}C_{22} - C_{12}^2>0$ and $C_{44}>0$)^42,43^, providing compelling evidence for the robust mechanical stability of PHOTH-G.

In Fig. 5a, the analysis of Young’s modulus in PHOTH-G uncovers slight anisotropic behavior in its deformation, stemming from the unique ring arrangement within its plane. The corresponding maximum Young’s Modulus values ($Y_{MAX}$) for deformations in the x and y directions are approximately 167.08 GPa and 161.02 GPa, respectively. These values are nearly one-tenth of the reported Young’s Modulus for graphene (1.0 TPa^44^). This substantial difference can be attributed to the intrinsic porosity of PHOTH-G, arising from the presence of 8-atom rings and the bond stiffness within fused tetragonal rings.

Figure 5b analyzes the Poisson ratio for PHOTH-G. In conventional materials, Poisson ratios typically fall within the range of 0.2 to 0.5^45^. A Poisson ratio 0.5 signifies incompressible materials, indicating minimal lateral dimension change under strain. Under uniaxial tensile loading in the x direction, PHOTH-G exhibits a maximum Poisson’s ratio ($\nu _{MAX}$) of 0.75, surpassing that observed in graphene (approximately 0.19^46^). This moderate Poisson ratio is attributed to the PHOTH-G lattice arrangement’s higher porosity than graphene. The increased porosity allows PHOTH-G to undergo more deformation under tension than graphene, resulting in this elevated value.

The slight mechanical anisotropy of PHOTH-G is further evident in Fig. 5b, where the minimum Poisson’s ratios ($\nu _{MIN}$) of about 0.21 and 0.35 occur under strains applied in the x-direction and y-direction, respectively. These values indicate relative incompressibility in these specific scenarios.

**Figure 6:**
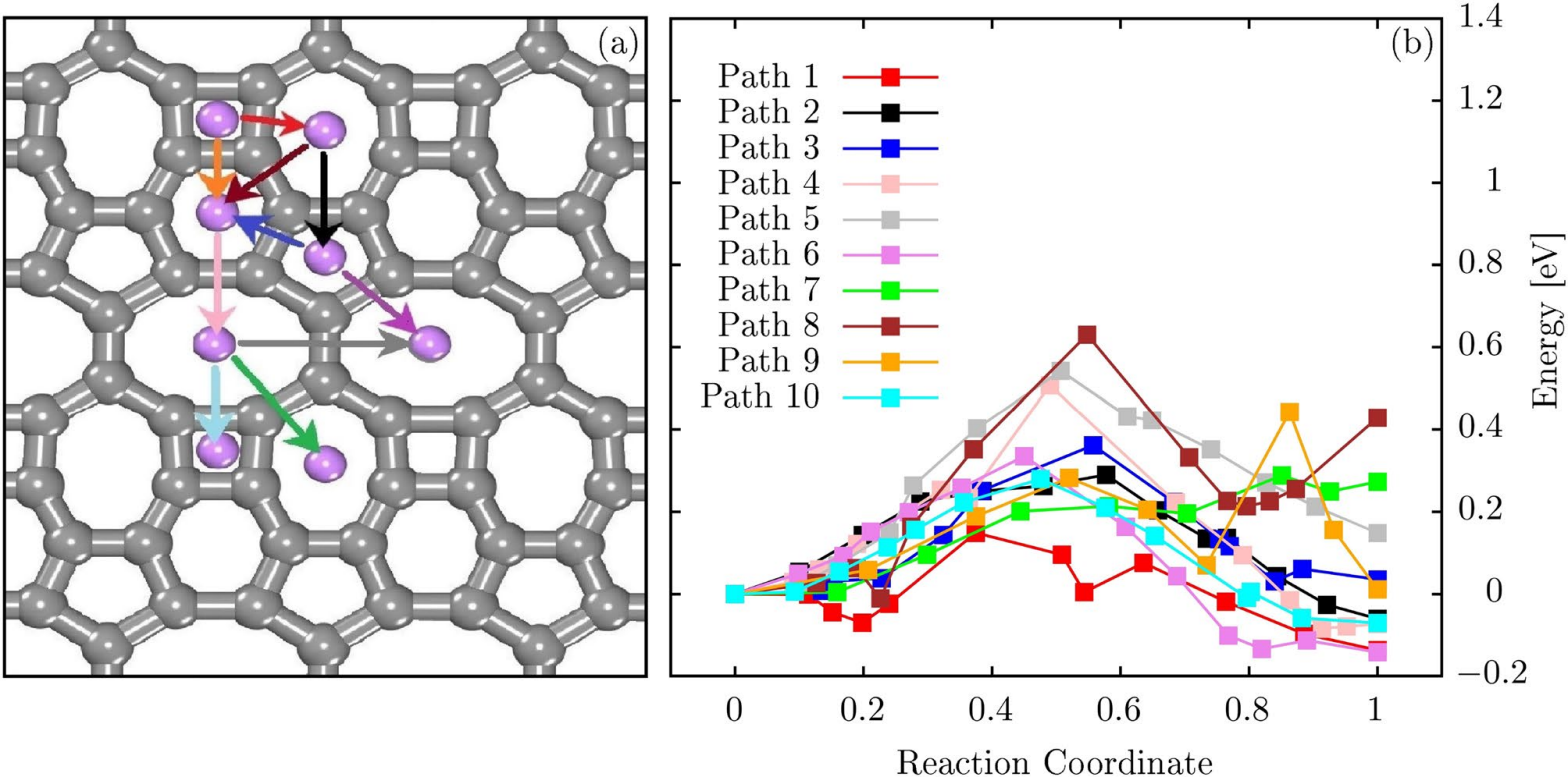
(**a**) Ten potential migration routes of a Li atom adsorbed in PHOTH-G and (**b**) their associated energy profiles. The arrow color in panel (**a**) is associated with the path color in panel (**b**).

Finally, we discuss the adsorption of lithium atoms on PHOTH-G as an example of its applicability. Figure 6a illustrates selected migration pathways of a lithium atom on the PHOTH-G surface, providing a visual representation of the potential diffusion routes. Figure 6b, in turn, depicts their related transition states and energy barriers. The supplementary material presents an AIMD video for the adsorption and diffusion of lithium atoms on PHOTH-G.

The barrier between neighboring 4- and 8-atom rings (depicted in cyan) is comparatively higher, measuring approximately 0.63 eV. On the other hand, the diffusion barriers along the remaining pathways vary from 0.14 to 0.54 eV. The red path, corresponding to migration between neighboring 7- and 6-atom rings, shows a lower diffusion barrier than observed in graphene (about 0.31 eV)^47^.

It is noteworthy that the diffusion barriers identified in PHOTH-G align with those observed in other 2D carbon-based allotropes. Notably, these barriers are significantly lower than those found in the biphenylene network (2.44 eV) and phagraphene (2.07 eV)^22^. On average, Li atoms within this material encounter a migration barrier of merely 0.38 eV. This low barrier suggests favorable Li-ion mobility, indicating a promising charge/discharge rate for PHOTH-G. Such characteristics are crucial for its potential application in energy storage devices.

## Methods

To understand the atomic-level behavior of PHOTH-G, we used two computational techniques: Density Functional Theory (DFT) and Ab initio Molecular Dynamics (AIMD) simulations. These calculations were performed using the CASTEP software package^48^.

We explored the electronic structure and stability of PHOTH-G using different exchange-correlation functionals within the generalized gradient approximation (GGA). These functionals included Perdew-Burke-Ernzerhof (PBE)^49^ and the hybrid Heyd-Scuseria-Ernzerhof (HSE06)^50^. We employed norm-conserving pseudopotentials to account for interactions between atomic nuclei and electrons. Additionally, we ensured the convergence of our calculations by setting an appropriate energy cutoff of 600 eV and convergence criteria of 1.0 $\times$ 10^-5^ eV. Forces on each atom were kept below 1.0 $\times$ 10^-3^ eV/Å.

To efficiently sample the electronic wavefunctions, we employed different k-point grids depending on the specific calculation. Geometric optimization and electronic property calculations used a denser grid of $10\times 10\times 1$ and $15\times 15\times 1$ for PBE, respectively, compared to $5\times 5\times 1$ used in HSE06 calculations due to the latter’s higher accuracy. The PHOTH-G unit cell has a vacuum region of 20Å adopted to prevent interactions between periodic images.

We analyzed the vibrational properties of PHOTH-G using a high-accuracy linear response method. Additionally, we performed AIMD simulations on a large supercell ($3\times 3\times 1$ ) containing 80 atoms to assess its long-term stability at a specific temperature. By employing a fixed time step of 1.0 fs over 5.0 ps, the temperature was controlled using the Nosé-Hoover thermostat^51^, in line with other AIMD studies^36,37^.

To investigate how lithium atoms interact with the surface of PHOTH-G, we incorporated van der Waals corrections within the Grimme scheme^52^. To determine the transition states for Li migration on the PHOTH-G surface, we first define the initial configuration with Li adsorbed on PHOTH-G as the reactants. Then, we identify the final positions of Li in neighboring rings relative to the initial configuration as the products. We employ two methods to identify these transition states: the linear synchronous transit (LST) and Quadratic Synchronous Transit (QST) methods^53^. LST involves a single interpolation to locate the maximum energy point. At the same time, QST combines searches for energy maxima with constrained minimizations to refine the transition state more accurately. Both methods are implemented in the TS Search module of Materials Studio. The outcomes of this module include the final product configuration and the associated energy barrier. Finally, we studied the optical properties of PHOTH-G under an applied electric field, utilizing a standard approach based on the complex dielectric constant^54^.

## Conclusions

We performed DFT and AIMD simulations to introduce PHOTH-G as a new 2D carbon allotrope family member. This material demonstrates programmable semiconducting properties with its porous topology comprising 4-5-6-7-8 carbon rings achieved through a bottom-up approach. Its low-energy structure ensures good dynamic, thermal, and mechanical stability. AIMD simulations reveal an intact lattice structure with no observable bond breaking or reconstructions at 1000 K.

The PHOTH-G metallic signature is characterized by electronic states with a reasonably higher degree of delocalization. This material has slight mechanical anisotropy with Young modulus and Poisson ratios varying between 7.08-167.8 GPa and 0.21-0.96, respectively. Its optical activity occurs in the visible and ultraviolet ranges. Li atoms adsorbed on the PHOTH-G surface encounter a migration barrier averaging 0.38 eV. This low barrier suggests advantageous Li-ion mobility, signifying a promising charge/discharge rate for PHOTH-G. This characteristic is crucial for its potential applications in energy storage devices.

### Supplementary Information

Supplementary Information 1.

Supplementary Information 2.

## Data Availability

Data supporting this study’s findings are available upon reasonable request from the last author L.A.R.J.
