# Supplementary material for: PHOTH-graphene: a new 2D carbon allotrope with low barriers for Li-ion mobility
Source: Sci Rep. 2024 Apr 25;14:9526. doi: 10.1038/s41598-024-59858-y (PMC11045837; doi:10.1038/s41598-024-59858-y)
Supplement: Supplementary file 1 — Supplementary Information 1. [file 41598_2024_59858_MOESM1_ESM.docx]

Title: Lithium diffusion on PHOTH-G.

Supplementary video: AIMD simulation for the adsorption and diffusion of lithium atoms on PHOTH-G.
